# Mosquitoes (Culicidae) as a vector of *Encephalitozoon hellem* (Microsporidia)

**DOI:** 10.1080/22221751.2024.2317914

**Published:** 2024-03-05

**Authors:** Artur Trzebny, Olena Nahimova, Natalia Volkova, Denys Hryhoriev, Anna Slodkowicz-Kowalska, Miroslawa Dabert

**Affiliations:** a Molecular Biology Techniques Laboratory, Faculty of Biology, Adam Mickiewicz University, Poznan, Poland; b Genetics and Cytology Department, School of Biology, V.N. Karazin Kharkiv National University, Kharkiv, Ukraine; c Department of Biology and Medical Parasitology, Faculty of Medicine I, University of Medical Sciences, Poznan, Poland

Microsporidiosis is an emerging infectious disease caused by eukaryotic parasites of the phylum Microsporidia. To date, the disease has been attributed to 17 species across eight genera, including one holding genus labelled as ‘*Microsporidium*’. These microorganisms exhibit diverse clinical manifestations [1]. Immunocompromised individuals are particularly vulnerable to these parasites. During infection, patients are at a risk of developing prostatitis, pneumonitis, nephritis, keratoconjunctivitis, sinusitis, urethritis, or cystitis. Microsporidians may also cause life-threatening chronic diarrhoea in immunocompromised patients [2]. The most common microsporidian infecting humans is *Enterocytozoon bieneusi*, followed by three *Encephalitozoon* species, including *E. hellem* [3]. *Encephalitozoon hellem* mainly exhibits distribution among birds and has been detected in avian tissues and faecal samples, particularly during the last decade in Europe. However, monkeys, bats, carnivores, and rodents may also be infected by this microsporidian [4]. Recently, preliminary reports have suggested the presence of *E*. *hellem* in mosquitoes [5]. Therefore, to address the question of the potential role of mosquitoes in transmitting this human microsporidiosis agent, we applied the recently developed DNA metabarcoding approach [5] to screen mosquito adults collected along a c.a. 2,000 km longitudinal transect in Europe Figure 1.

**Figure 1.**
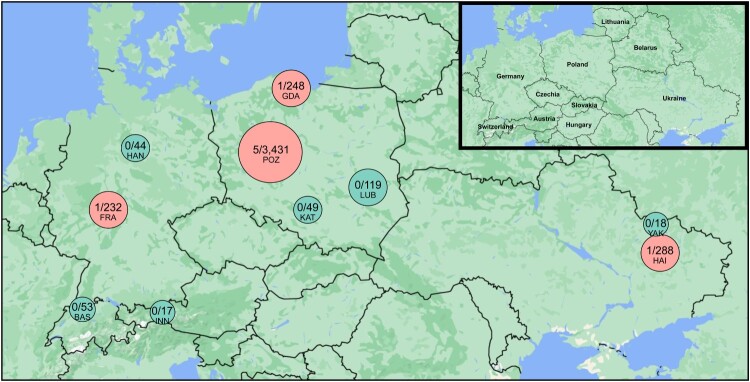
Sampling places of mosquitoes with (red) and without (green) *Encephalitozoon hellem*-positive individuals. The size of circle corresponds to the number of analysed mosquitoes. Numeric value inside the circles: number of microsporidian-positive individuals/number of analysed mosquitoes. BAS: Basel; FRA: Frankfurt; GDA: Gdansk; HAI: Haidary; HAN: Hannover; INN: Innsbruck; KAT: Katowice; LUB: Lublin; POZ: Poznan; YAK: Yakovlivka.

In total, 4,503 mosquitoes, including 2,565 females and 1,938 males, collected from 10 cities located in five European countries were individually screened for microsporidian DNA (Figure; Appendix Table 1) using the metabarcoding method described by Trzebny et al. [5]. This method enables the detection of all microsporidian species, including those that are pathogenic to humans. Briefly, fragments of the cytochrome c oxidase subunit I gene (mini-COI) and the hypervariable V5 region of the 18S rRNA gene were amplified and sequenced to identify the mosquito and microsporidian species, respectively (Appendix Table 2). Bioinformatics analysis was conducted using a custom workflow in Geneious Prime version 2023.2.1 (Biomatters Ltd.), USEARCH version 11, and Qiime2 version 2023.9. Presence of *E. hellem* was confirmed through quantitative PCR (qPCR) using the polar tube protein 1 gene (PTP1) as a target. The Luna Universal Probe qPCR Master Mix (NEB, USA) with E.hell-PTP_F (AATGACGCCGGGTGTTTCTC) and E.hell-PTP_R (GTCCTGGCTGGCATGGATAA) primer sets and E.hell-PTP_P (GGGGATGGAAGCAACCAGAC) TaqMan probes developed in this study were used. The genotype of *E. hellem* was determined by sequencing the almost complete 18S rRNA gene [6] and approximately 1,200 bp of the PTP gene [7] (Appendix Table 2). Phylogenetic trees were constructed based on the published 18S rRNA and PTP sequences representing all known *E. hellem* genotypes (Appendix Table 3) [7]. Trees were reconstructed using maximum likelihood through GARLI and Bayesian inference using MrBayes software, as described by Trzebny et al. [8]. The specificity of infection against the sites was calculated using the Kruskal–Wallis test. The sequences generated in this study were deposited in GenBank under the accession numbers OR780779–OR780786 and OR789801–OR789808 (Appendix Table 3).

Based on the mini-COI sequence data, all the mosquitoes were unambiguously assigned to 14 species belonging to six genera (Appendix Table 4). *Encephalitozoon hellem* DNA was identified in eight female mosquitoes (0.03%; 95% CI: 0.002–0.006) collected from Germany (1/175), Poland (6/2,161), and Ukraine (1/193) (Figure; Appendix Table 1). Microsporidian DNA has been found in various mosquito species, including *Aedes vexans* (3), *Ochlerotatus cantans* (3), *Annopheles messeae* (1), and *Culex pipiens* (1) (Appendix Table 5). Presence/absence qPCR analysis confirmed the presence of *E. hellem* in all samples that were positive in the metabarcoding method (1.190 ⩽ ΔRn ⩽ 1.237; Appendix Table 6). The presence of *E. hellem* in mosquitoes was not locally limited (*p* = 0.392). Phylogenetic analysis of 18S rRNA and PTP1 sequences clustered *E. hellem* within the human pathogenic genotype 1A (Appendix Figure). Previously, genotype 1A has been recorded in humans and companion birds [4,7].

To date, only three microsporidian species (*Anncaliia algerae*, *Tubulinosema acridophagus*, and *Trachipleistophora hominis*) have been identified in culicids [9]. Our results suggest that as many as one in 200 mosquitoes can transmit another microsporidiotic agent, *E*. *hellem*. This suggests that mosquito species represented in this study by low numbers of individuals may also transmit *E*. *hellem* (Appendix Table 4). Notably, we did not detect any other human pathogenic microsporidians in the tested mosquitoes [8]. This suggests that *E*. *hellem* is the most common microsporidian species infecting wild vertebrates. The presence of *E*. *hellem* only in female mosquitos clearly indicates that they acquire this microsporidian during feeding and that the microsporidian DNA originates from spores released into the blood of infected vertebrates. Human infection can occur through the mosquito saliva during anesthesia of the bite or, more likely, may be a result from crushing an infected female mosquito, thereby mechanically inoculating the spores into a skin-bite wound [10]. However, these potential transmission mechanisms are speculative and require additional detailed research. Our study provides preliminary data for the future surveillance of mosquito-borne microsporidians and highlights the need for further studies to decipher the transmission dynamics of these parasites. Moreover, clinical awareness of this parasitic infection should be strengthened through educational and interdisciplinary collaboration between clinicians and parasitologists.

## Supplementary Material

Appendix

## References

[1] Han B, Weiss LM. Microsporidia: obligate intracellular pathogens within the fungal kingdom. Microbiol Spectr. 2017 Mar 10;5(2). 10.1128/microbiolspec.funk-0018-2016 PMC5613672 28944750

[2] Weiss LM, Schwartz DA. Tropical infectious diseases: principles, pathogens and practice. Edinburgh: Elsevier Inc.; 2011. 714–721 p.

[3] Ruan Y, Xu X, He Q, et al. The largest meta – analysis on the global prevalence of microsporidia in mammals, avian and water provides insights into the epidemic features of these ubiquitous pathogens. Parasit Vectors. 2021;14(1):1–14. 33794979 10.1186/s13071-021-04700-x PMC8017775

[4] Hinney B, Sak B, Joachim A, et al. More than a rabbit’s tale - Encephalitozoon spp. in wild mammals and birds. Int J Parasitol Parasites Wildl. 2016;5:76–87. 28560162 10.1016/j.ijppaw.2016.01.001 PMC5439460

[5] Trzebny A, Slodkowicz-Kowalska A, Becnel JJ, et al. A new method of metabarcoding Microsporidia and their hosts reveals high levels of microsporidian infections in mosquitoes (Culicidae). Mol Ecol Resour. 2020 Nov;20(6):1486–1504. 32516485 10.1111/1755-0998.13205 PMC7818484

[6] Weiss LM, Zhu X, Cali A, et al. Utility of microsporidian rRNA in diagnosis and phylogeny: A review. Folia Parasitol (Praha). 1994;41(2):81–90. 7927064

[7] Xiao L, Li L, Moura H, et al. Genotyping Encephalitozoon hellem isolates by analysis of the polar tube protein gene. J Clin Microbiol. 2001 Jun;39(6):2191–2196. 11376056 10.1128/JCM.39.6.2191-2196.2001 PMC88110

[8] Trzebny A, Mizera J, Dabert M. Microsporidians (Microsporidia) parasitic on mosquitoes (Culicidae) in central Europe are often multi-host species. J Invertebr Pathol. 2023 Mar;197:107873. 36577478 10.1016/j.jip.2022.107873

[9] Seatamanoch N, Kongdachalert S, Sunantaraporn S, et al. Microsporidia, a highly adaptive organism and Its host expansion to humans. Front Cell Infect Microbiol. 2022 Jun 16;12:924007. 35782144 10.3389/fcimb.2022.924007 PMC9245026

[10] Coyle CM, Weiss LM, Rhodes L V, et al. Fatal myositis due to the microsporidian brachiola algerae, a mosquito pathogen. N Engl J Med. 2004 Jul;351(1):42–47. 15229306 10.1056/NEJMoa032655 PMC3109631

