# Supplementary material for: Mosquitoes (Culicidae) as a vector of Encephalitozoon hellem (Microsporidia)
Source: Emerg Microbes Infect. 2024 Mar 5;13(1):2317914. doi: 10.1080/22221751.2024.2317914 (PMC10916917; doi:10.1080/22221751.2024.2317914)
Supplement: Appendix [file TEMI_A_2317914_SM2095.pdf]

Mosquitoes (Culicidae) as a vector of *Encephalitozoon hellem* (Microsporidia)

Appendix

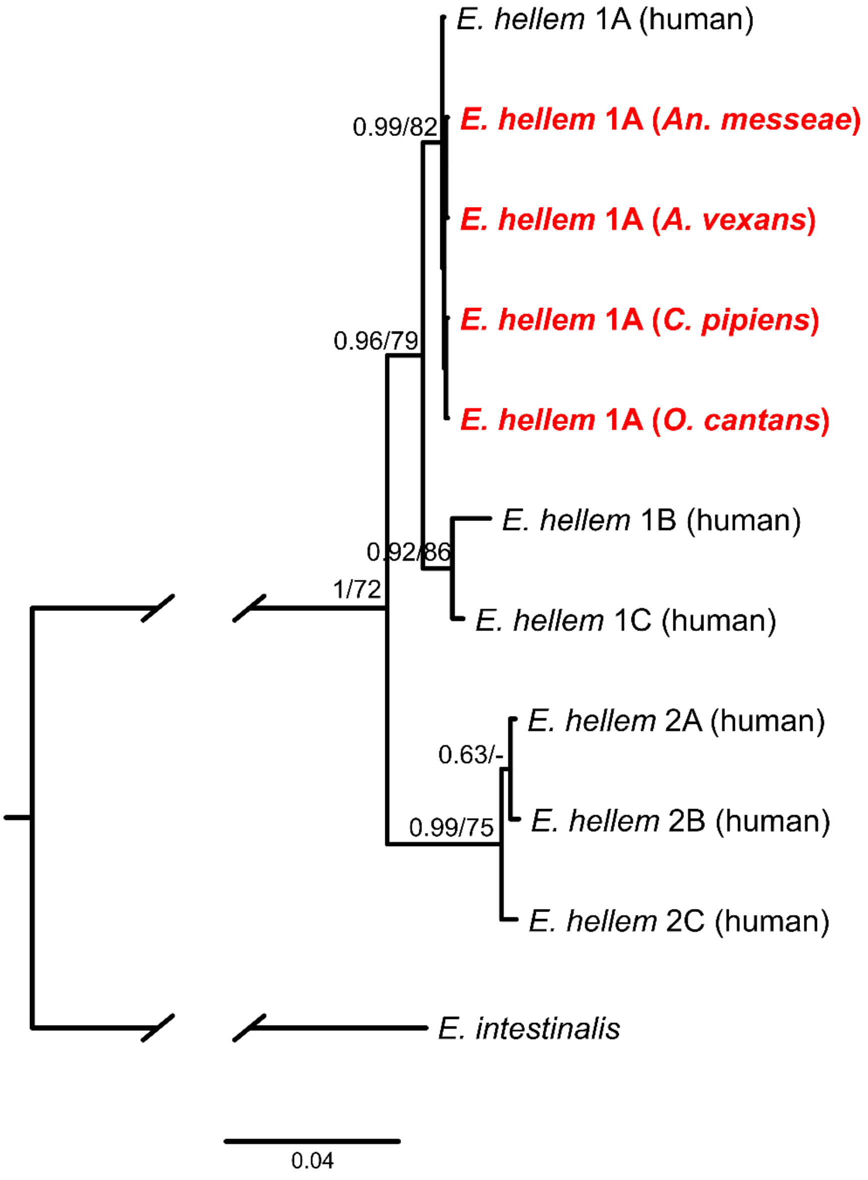

Appendix Figure. Phylogenetic tree of *Encephalitozoon hellem* inferred from BI and ML analyses of concatenated 18S rRNA and polar tube protein gene sequence data. Values near the branches show Bayesian posterior probabilities (PP) and bootstrap support values (BS) (PP/BS). The sequences identified in this study are shown in red and bold.

Appendix Table 1. Number of mosquitoes collected from each study area. Values in parentheses indicate the number of individuals positive for *Encephalitozoon hellem*.

Study area	Female		Male	
	Value	95% CI	Value	95% CI
Austria, Innsbruck	17	0.000 – 0.000	0	0.000 – 0.000
Germany, Frankfurt	143 (1)	0.001 – 0.039	89	0.000 – 0.000
Germany, Hannover	32	0.000 – 0.000	16	0.000 – 0.000
Poland, Gdansk	185 (1)	0.001 – 0.030	63	0.000 – 0.000
Poland, Katowice	42	0.000 – 0.000	7	0.000 – 0.000
Poland, Lublin	74	0.000 – 0.000	45	0.000 – 0.000
Poland, Poznan	1860 (5)	0.001 – 0.006	1571	0.000 – 0.000
Switzerland, Basel	19	0.000 – 0.000	34	0.000 – 0.000
Ukraine, Haidary	175 (1)	0.001 – 0.032	113	0.000 – 0.000
Ukraine, Yakovlivka	18	0.000 – 0.000	0	0.000 – 0.000
**TOTAL**	**2565 (8)**	**0.002 – 0.006**	**1938**	**0.000 – 0.000**
	**4503 (8) [95% CI: 0.001 – 0.004]**			

Appendix Table 2. PCR primers used in this study.

Target	Primer	Sequence	Reference
SSU (V5 region)	CM-V5F	GATTAGANACCNNNGTAGTTC	(1)
	CM-V5R	TAANCAGCACAMTCCACTC	
SSU	V1	CACCAGGTTGATTCTGCCTGAC	(2)
	ss1492r	GGTTACCTTGTTACGACTT	
PTP	PTP_F	ATGAAAGGTATTTCGAAGAT	(3)
	PTP_R	GCCTCCATGGCATACTGC	

Appendix Table 3. GenBank accession numbers for DNA sequences used in the *Encephalitozoon hellem* phylogenetic analyses.

Species	Genotype	Host	GenBank no.	
			18S rRNA gene	PTP
Encephalitozoon hellem	1A	*Homo sapiens*	AF338365.1	AF338361.1
Encephalitozoon hellem	1B	*Homo sapiens*	L13331.1	AY024342.1
Encephalitozoon hellem	1C	*Homo sapiens*	AF338366.1	AF338362.1
Encephalitozoon hellem	2A	*Homo sapiens*	AF110327.1	n/a
Encephalitozoon hellem	2B	*Homo sapiens*	AF338364.1	AF338363.1
Encephalitozoon hellem	2C	*Homo sapiens*	AF110328.1	n/a
Encephalitozoon hellem	1A	*Aedes vexans*	OR780779.1	OR789801.1
Encephalitozoon hellem	1A	*Annopheles messeae*	OR780782.1	OR789804.1
Encephalitozoon hellem	1A	*Culex pipiens*	OR780783.1	OR789805.1
Encephalitozoon hellem	1A	*Ochlerotatus cantans*	OR780784.1	OR789807.1
Encephalitozoon intestinalis	n/a	*Homo sapiens*	CP075163.1	CP075163.1

Appendix Table 4. Number of mosquito species analysed. Values in parentheses indicate the number of individuals positive for *Encephalitozoon hellem*.

Study area	Female		Male	
	**Value**	**95% CI**	**Value**	**95% CI**
Aedes cinereus	47	0.000 – 0.000	5	0.000 – 0.000
Aedes geniculatus	4	0.000 – 0.000	0	0.000 – 0.000
Aedes vexans	494 (3)	0.000 – 0.000	352	0.000 – 0.000
Annopheles messeae	58 (1)	0.003 – 0.091	5	0.000 – 0.000
Coquillettidia richiardii	205	0.000 – 0.000	106	0.000 – 0.000
Culex hortensis	19	0.000 – 0.000	34	0.000 – 0.000
Culex pipiens	338 (1)	0.001 – 0.017	266	0.000 – 0.000
Culex territans	19	0.000 – 0.000	8	0.000 – 0.000
Culiseta annulata	11	0.000 – 0.000	0	0.000 – 0.000
Ochlerotatus annulipes	529	0.000 – 0.000	433	0.000 – 0.000
Ochlerotatus cantans	592 (3)	0.002 – 0.015	455	0.000 – 0.000
Ochlerotatus communis	59	0.000 – 0.000	48	0.000 – 0.000
Ochlerotatus punctor	77	0.000 – 0.000	106	0.000 – 0.000
Ochlerotatus sticticus	113	0.000 – 0.000	120	0.000 – 0.000
Aedes cinereus	47	0.000 – 0.000	5	0.000 – 0.000
**TOTAL**	**2565 (8)**	**0.002 – 0.006**	**1938**	**0.000 – 0.000**
	**4503 (8) [95% CI: 0.001 – 0.004]**			

Appendix Table 5. Number of individuals of each mosquito species collected from each study area. Values in parentheses indicate the number of individuals positive for *Encephalitozoon hellem*.

Country	City	Species	Female	Male
Austria	Innsbruck	*Aedes cinereus*	12	0
Austria	Innsbruck	*Aedes geniculatus*	4	0
Austria	Innsbruck	*Ochlerotatus cantans*	1	0
Germany	Frankfurt	*Culex pipiens*	76 (1)	26
Germany	Frankfurt	*Ochlerotatus annulipes*	32	41
Germany	Frankfurt	*Ochlerotatus cantans*	25	18
Germany	Frankfurt	*Ochlerotatus sticticus*	10	4
Germany	Hannover	*Aedes cinereus*	8	5
Germany	Hannover	*Ochlerotatus cantans*	21	11
Germany	Hannover	*Ochlerotatus punctor*	3	0
Poland	Gdansk	*Aedes vexans*	33	13
Poland	Gdansk	*Annopheles messeae*	44	5
Poland	Gdansk	*Coquillettidia richiardii*	45	0
Poland	Gdansk	*Ochlerotatus cantans*	63 (1)	45
Poland	Katowice	*Aedes vexans*	31	4
Poland	Katowice	*Culex pipiens*	4	3
Poland	Katowice	*Ochlerotatus cantans*	7	0
Poland	Lublin	*Aedes cinereus*	13	0
Poland	Lublin	*Aedes vexans*	11	15
Poland	Lublin	*Culex pipiens*	8	16
Poland	Lublin	*Ochlerotatus annulipes*	25	6
Poland	Lublin	*Ochlerotatus cantans*	15	0
Poland	Lublin	*Ochlerotatus punctor*	2	8
Poland	Poznan	*Aedes cinereus*	13	0
Poland	Poznan	*Aedes vexans*	266 (2)	210
Poland	Poznan	*Annopheles messeae*	14 (1)	0
Poland	Poznan	*Coquillettidia richiardii*	143	106
Poland	Poznan	*Culex pipiens*	250	218
Poland	Poznan	*Culex territans*	19	8
Poland	Poznan	*Culiseta annulata*	11	0
Poland	Poznan	*Ochlerotatus annulipes*	472	386
Poland	Poznan	*Ochlerotatus cantans*	445 (2)	381
Poland	Poznan	*Ochlerotatus communis*	59	48
Poland	Poznan	*Ochlerotatus punctor*	72	98
Poland	Poznan	*Ochlerotatus sticticus*	96	116
Switzerland	Basel	*Culex hortensis*	19	34
Ukraine	Haidary	*Aedes vexans*	135 (1)	110
Ukraine	Haidary	*Aedes cinereus*	1	0
Ukraine	Haidary	*Coquillettidia richiardii*	17	0
Ukraine	Haidary	*Culex pipiens*	0	3
Ukraine	Haidary	*Ochlerotatus cantans*	15	0
Ukraine	Haidary	*Ochlerotatus sticticus*	7	0
Ukraine	Yakovlivka	*Aedes vexans*	18	0

Appendix Table 6. Delta Rn (ΔRn) values measured during *Encephalitozoon hellem* detection in each individual positive for *E. hellem*.

Sample	Species	Country	City	ΔRn		
				First replicate	Second replicate	Mean
Mosquito	*Aedes vexans*	Poland	Poznan	1.199	1.243	1.221
Mosquito	*Aedes vexans*	Poland	Poznan	1.193	1.222	1.207
Mosquito	*Aedes vexans*	Ukraine	Haidary	1.229	1.245	1.237
Mosquito	*Annopheles messeae*	Poland	Poznan	1.212	1.223	1.217
Mosquito	*Culex pipiens*	Germany	Frankfurt	1.204	1.179	1.191
Mosquito	*Ochlerotatus cantans*	Poland	Gdansk	1.180	1.199	1.190
Mosquito	*Ochlerotatus cantans*	Poland	Poznan	1.184	1.209	1.197
Mosquito	*Ochlerotatus cantans*	Poland	Poznan	1.196	1.209	1.202
Control (-)	-----	-----	-----	0.352	0.354	0.353

References

1. Trzebny A, Slodkowicz-Kowalska A, Becnel JJ, Sanscrainte N, Dabert M. A new method of metabarcoding Microsporidia and their hosts reveals high levels of microsporidian infections in mosquitoes (Culicidae). Mol Ecol Resour. 2020 Nov;20(6):1486–504.
2. Weiss LM, Zhu X, Cali A, Tanowitz HB, Wittner M. Utility of microsporidian rRNA in diagnosis and phylogeny: A review. Folia Parasitol (Praha). 1994;41(2):81–90.
3. Xiao L, Li L, Moura H, Sulaiman I, Lal AA, Gatti S, et al. Genotyping Encephalitozoon hellem Isolates by Analysis of the Polar Tube Protein Gene. J Clin Microbiol. 2001 Jun;39(6):2191–6.
